# Gossamer

**Published:** 1834

**Authors:** 


### Art. III.—Gossamer.

Having *administered* to our readers in one *unbroken dose*, the *officinal* report of the proceedings of the late Medical Convent on at Columbus, without as yet seeing any scowling movements of the seventh pair of nerves, we are encouraged to *exhibit* a second, promising not to repeat it for three years. By the end of that long *period*, the *vis medicatrix naturæ*, will, we hope, have restored them from the influence of the present *portions;* especially, as that we are about to give, will be a *diffusible stimulus*, and of course, altogether transient in its effects.

We happened once to know a President—not of the United States, for they are not easily *known*, but of a College—not the College of Pharmacy neither, but of the arts and sciences, (not meaning however that pharmacy is deficient in either,) —who happened to introduce a new word into one of his daily orisons, and was immediately rewarded with the clappings of his pupils. But what of that? Why—the doctors of Ohio have had a meeting—it ran through half a week—occupied itself on a variety of subjects, (excluding professorships, patients and fees,) and parted without a quarrel, or even the slightest *nervous irritation*. They deserve applause then, and let them have the clapping that is due from those, who take delight in declaiming against our respectable profession.

Ovid informs us, that the Alcedo had the power of calming the waves of the sea; but as the King-fisher is not in this climate through the winter, our Halcyon days cannot be ascribed to his pacific influence; and although a different aquatic bird was present, it is well known, that his power consists in raising, instead of tranquillizing the *breakers*. It is more probable then, as the *expressed oils* can quiet the agitations of the ocean, that the different organs of our body, might have been so lubricated as to move smoothly over one another. Whatever theory may be adopted, we can testify, that the meeting was altogether a most harmonious and pleasant affair. The 10 or 12 committees, composed of gentlemen who are accustomed to *despatch subjects*, were ready, and actually did report, on the second and third days of the Convention; and although all the reports were not as long, they had the merit of being as much to the point, as those made in some other general assemblies. And then for the disucssions. Aye, they might have been studied as a model. No *caustic* applications to a worthy, but timid and flinching brother, to make him wince, for the diversion of his opponents; no long and limping speeches, to *narcotise* the Convention; no anxious hopes and fears about the temper of constituents; no concern as to the kind of *political* epidemic, which may prevail on the second Tuesday of October next; no calling for the yeas and nays, on a *rider* of seven words, upon the seventh amendment, to the seventh resolution, of a series, declaratory of some old medical axiom. But, on the contrary, a "small chance" of technicalities; a "powerful sight" of brevity; a "mighty" straight-forwardness; an early *crisis*, and a speedy call for the final vote, demonstrated the absence of every kind of monied influence, the *radix* of "lengthy" debates, as well as of many other lesser evils.

Some of the members regretted, that the convention had not been called for some other month than January; but if that body would render its good example beneficial to others, it should continue its meetings, when those who need an exemplar in deliberative proceedings, are in session.

Its *modus operandi* led to other, and, as it respects the profession, more interesting fruits. Several old friends met for the first time for many years; and found that, by having lived apart, they were as good friends as ever; others, who had long corresponded, formed a personal acquaintance; some who had cherished prejudices against others, had their minds *depurated;* pupils and preceptors met as equals, and exchanged pledges to meet again; the *callidum innatum* of every heart was raised above mean heat; and even the temperature of the atmosphere; was kept quite comfortable while the *tinctures* froze in the bottles of our isolated and unsocial brethren, east of the mountains.

We hope that the *excitement* of which we speak, will spread

*sympathetically* through all the *organs* of which our profession is composed; that medical conventions will speedily be gotten up in all the states, and especially those of the West, where the *organization* is still so incomplete, from the constant *infiltration* of of new *elements.* Let them not consist of delegates, nor be bound together by the *twisted sutures* or *many tailed bandages* of legislation; but primary assemblies, for social intercourse, mutual encouragement, deliberation, and declaratory reports and resolutions, on the subjects, which should occupy the attention of the members of a scientific and benevolent profession. We should even delight to see it become fashionable for the physicians of neighboring states, to visit each other on these occasions; and hope, (if preserved from the hands of the *doctors* and druggists,) to live long enough to participate in such *consultations.*

The West is dashing on like a steamboat. The *nutritive secretion* has great activity, and every day sets up some now mode of *organization.* There is even danger of *hypertrophy*, before it has acquired mature age; but medical men are not accessary to this effect, except by preserving too many lives; and they should not hesitate to unite themselves into one *organ;* and attract to it a full share of the *nutritive matter*, which *circulates* through the great social body; for otherwise, they may lose their *vitality* and *slough* off.

At night, after the convention adjourned, a majority of the members met together, for a farewell greeting; and brethren from the valley of the Cuyhoga, the Muskingum, the Sciota and the Miamies, exchanged reciprocal expressions of good feeling, and laid plans for future correspondence, and social visitings—confirming their promises with a glass of Madeira, instead of *antimonial*, wines reserved in charity for their patients.

When the hour of parting arrived, one of them favoured the company with a song, prepared for the occasion; and as the lyrick department of our *medicinal* literature (containing dirges only) is rather in a state of *atrophy*, we shall make it the gossamer of this badinage.

### PARTING SONG.

### Tune—Auld Lang Syne.

I.

From Erie's chill and misty coast,
Ohio's sunnier shore,
We came to blend our various thoughts,
Then part to meet no more.
Ah! we must part my stranger-friends,
This kindly greeting o'er,
But let our hearts together cling,
Deep pledg'd forevermore.

II.

As round the festive board we stand,
Where wit and song combine,
Let gen'rous glances mingle bright,
And soul with soul entwine:
For soul with soul, my trusty friends,
When mingling deep and warm,
Can glow beneath the world's cold frown,
And mock its cruel scorn.

III.

When o'er the midnight lamp we toil,
Nor mark the fleeting hours,
The dear remembrance of this night
Will cheer our languid pow'rs.
Your notes of melody, my friends,
Will swell and echo round,
And wake our dull and dreamy thoughts
With soul inspiring sound.

IV.

When mercy's voice shall call us forth—
The storm-fire for our light—
The tempest-song shall welcome bring
The music of this night.
Then, high and strong, my social friends,
Our glad'nened hearts will beat,
And swifter on the path will fall,
Our light and bounding feet.

V.

And while beside the bed of death,
In darkness and despair,
We soothe the wretched, friendless man,
To fancy you'll appear

A faithful band of happy friends,
To light the dismal gloom,
With kindling eyes, like torches round
The dark and dreary tomb.

VI.

If near the trav'ler's blazing fire,
We meet in foreign lands,
The tear of friendship, warm, will fall
Upon our clasping hands.
Then shall this parting song, my friends,
Ring gaily through the dome,
And sweeping o'er the heart's soft lyre,
Recall the joys of home.

VII.

And when old time around our brows
Shall bind the snowy wreath,
Bright visions of this scene shall rise,
To stay the hand of death;
For Auld Lang Syne, my friends,
With genial, glowing ray,
Will 'mid the gath'ring clouds of age,
Revive this happy day.

